# Exosomal miR-93-5p regulated the progression of osteoarthritis by targeting ADAMTS9

**DOI:** 10.1515/med-2023-0668

**Published:** 2023-03-15

**Authors:** Yachen Li, Junjun Duan, Weicheng Lin, Jie Liu

**Affiliations:** Department of Orthopedics, The First People’s Hospital of Yunnan Province, Yunnan, China; Department of Plastic Surgery, Central Hospital of Wuhan, Huazhong University of Science and Technology, Wuhan, Hubei, China

**Keywords:** osteoarthritis, miR-93-5p, ADAMTS9, autophagy, apoptosis

## Abstract

Osteoarthritis (OA) is a type of common degenerative joint disorder, in which adipose mesenchymal stem cells (ADSCs) and the secreted exosomes play an important role. The purpose of this study was to investigate the role and mechanism of exosomes derived from ADSCs (ADSC-exos) in OA. The gradient of IL-1β concentration was designed to construct the articular chondrocyte model of arthritic mice. The expression of miR-93-5p and ADAMTS9 in articular chondrocytes was detected by reverse transcription quantitative polymerase chain reaction. Dual luciferase reporter gene assay was performed to verify the interaction between them. Monodansylcadaverine staining was used to visualize the autophagosome formation and cell apoptosis was analyzed by flow cytometry. ADSC-exos were authenticated by transmission electron microscope and western blot assay. miR-93-5p was found to be downregulated in IL-1β-treated articular chondrocytes compared with OA cartilage while ADAMTS9 was upregulated, which was identified as a direct target gene of miR-93-5p. Silencing of ADAMTS9 attenuated the effects of miR-93-5p. Exosomal miR-93-5p can reduce the release of inflammatory factors in mouse arthritis cell models. This study first described the mechanism under that ADSC-exos inhibited inflammation and alleviated OA through the innovative targets miR-93-5p/ADAMTS9 signal axis. This provided a new method for the treatment of OA.

## Graphical abstract

**Figure:**
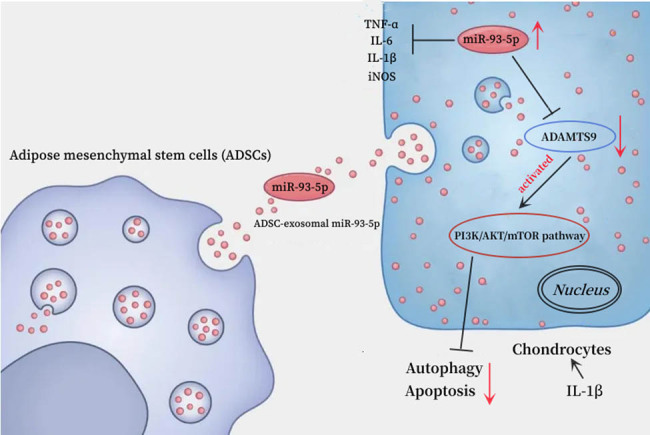

## 1 Introduction

Osteoarthritis (OA), a frequently seen degenerative joint disorder, is associated with gradual degradation of articular cartilage, inflammation, and joint malfunction [1,2]. OA displays the consistent degeneration of articular cartilage, causing the imbalanced generation and degeneration of articular chondrocyte extracellular matrix [3]. At present, the clinical treatment for this disease mainly includes non-steroidal anti-inflammatory drugs or joint replacement surgery [4]. It is significant to elucidate the pathogenesis of OA and search for a potential treatment strategy that avoids surgery.

In OA, many inflammatory cytokines, tumor necrosis factor (TNF)-α and interleukin 1 (IL-1), are produced by the synovium and the chondrocytes [5,6]. In OA, the dynamic balance of adjustment mechanism transformation is broken, which is manifested by the generation of the aggrecan [7], as well as type II collagen being depleted [8], while the production of matrix-degenerating enzymes (matrix metalloproteinases [MMPs]) is reinforced [9]. Among them, the protease (aggrecanase) enzymes encoded by ADAMTS are called ADAMTS enzymes [10]. ADAMTS family contains 20 members, and the studies have put forward that the ADAMTS 1, 4, 5, 8, 9, and 15 genes have aggrecanase activities [11]. The analysis of gene expression and methylation datasets identified ADAMTS9 as the biomarkers for OA [12]. Other molecular mechanisms include the change in mediators of apoptosis polymerase and modification of homeostatic processes, which included autophagy [13,14,15]. The research showed that chondrocyte autophagy, as a self-protective mechanism, can recuperate chondrocytes’ viability, while apoptosis of chondrocytes may lead to the degeneration and failed regeneration of the cartilage, which is considered a potential target for regulating the progression of OA [16,17]. Furthermore, OA therapies appear far from satisfactory.

Widely studies found that abnormal gene expression in OA articular chondrocytes leads to its pathogenesis [18]. Recently, microRNAs (miRNAs) are frequently dysregulated in human inflammatory diseases including OA, which possess epigenetic-like properties in the regulation of gene expression after transcription [19,20]. miRNAs play a key role in a variety of biological processes by targeting its 3′-UTR region to inhibit mRNAs’ expression, thereby blocking its translation process or inducing cleavage [21]. For example, miR-103 contributed to OA development by directly targeting and inhibiting the expression of Sox6, which may be an effective treatment for OA [22]. The study also found that miR-375 can inhibit the expression of ATG2B in chondrocytes, suppress autophagy, and promote the endoplasmic reticulum stress [23]. Thus, we inferred that miRNAs play a crucial role in the development of arthritis and joint homeostasis and that their mechanisms of action in the progression of OA may contribute to improving the efficacy of OA treatment.

miR-93-5p is a member of an miRNA family, which is associated with inflammatory diseases [24]. Previous study found that miR-93-5p is involved in the inflammatory and anti-proliferation processes of neodymium oxide-induced human bronchial epithelial cell lines by targeting Nd_2_O_3_ [25]. A study also confirmed that miR-93-5p-enhanced adipose mesenchymal stem cell (ADSC)-derived exosomes can prevent cardiac injury by targeting Atg7 and Toll-like receptor 4 (TLR4) and inhibiting hypoxia-induced autophagy and inflammatory cytokine expression [26]. Furthermore, miR-93-5p has been revealed to regulate IL-1β-induced cartilage degradation and chondrocyte apoptosis in OA by targeting TCF4 [27]. However, the role of miR-93-5p in OA remains to be elucidated.

Recent studies have shown that exosomes derived from adipose mesenchymal stem cells (ADSC-exos) play an important role in OA [28,29]. For example, exosomes from ADSCs promote chondrogenesis and suppress inflammation by upregulating miR-145 and miR-221 [30]. Although miR-93-5p has been found to be involved in the regulation of disease progression in a number of studies, the regulatory mechanism of miR-93-5p derived from ADSC exosomes in OA remains unclear.

To elucidate the potential role of ADSC-derived exosomal miR-93-5p in OA, we investigated the expression of miR-93-5p in cell models and analyzed the relationships among apoptosis, inflammation, and autophagy of miR-93-5p by targeting ADAMTS9. These data may provide new evidence that exosomes derived from ADSCs have better therapeutic effects on OA.

We present the following article/case in accordance with the CONSORT reporting checklist.

## 2 Methods

### 2.1 Cell lines and treatments

Chondrocytes (Lot. 339995, Beina Biology Research Institute, China) were purchased and maintained in Dulbecco’s modified eagle medium containing 10% fetal bovine serum (FBS) and 1% penicillin and streptomycin (Gibco, USA). ADSCs were obtained from Procell Life Science & Technology Co., Ltd. (Wuhan, China) and cultured in mouse adipose mesenchymal stem cell complete medium (Procell, China). All media were supplemented with 10% FBS (Gibco, USA) and 1% penicillin/streptomycin. All cells were cultured in a humidified incubator at 37°C with 5% CO_2_. To construct an *in vitro* model of inflammation, chondrocytes treated for 2 h with IL-1β, which was acquired from R&D Systems (St. Paul, MN, USA).

### 2.2 Transient transfection assay

The ADAMTS9 short-interfering RNA (siRNA) came from GenePharma (China). The miR-93-5p mimic, inhibitor, and negative control (NC) oligonucleotides were purchased from Ribobio (China). Chondrocytes were transfected with them using Lipofectamine 2000 (Thermofisher, USA), according to the manufacturer’s instruction.

### 2.3 RNA extraction and reverse transcription-quantitative polymerase chain reaction (RT-qPCR)

According to the manufacturer’s instructions, TRIzol kit (Tiangen Biotech (Beijing, China) Co., Ltd.) was used to separate total RNA from glioma cells. Thereafter, UEIris RT-qPCR System for First-Strand cDNA Synthesis (with Dnase I) (YuhengBio Suzhou, China) and miRNA First-Strand cDNA Synthesis were used. Tail Addition Kit (Sangon Biotech Shanghai, China) was applied to reversely transcribe the RNA into cDNA, and SYBR 2* qPCR Master Mix-qPCR was used to perform PCR (YuhengBio) with glyceraldehyde 3‐phosphate dehydrogenase (GAPDH) adopted for endogenous control. Comparative quantification was detected using the 2^−ΔΔCt^ method. The sequences of the primers were as follows: GAPDH forward, 5′-AATGGTGAAGGTCGGTGTGA-3′; GAPDH reverse, 5′-CGTGAGTGGAGTCATACTGGAA-3′; TNF-α forward, 5′-CTCATTCCTGCTTGTGGC-3′; TNF-α reverse, 5′-TGTGAGTGTGAGGGTCTGG-3′; iNOS forward, 5′-CCTGCTTTGTGCGAAGTGTC-3′; iNOS reverse, 5′-CCCAAACACCAAGCTCATGC-3′; IL-6 forward, 5′-TCGTGGAAATGAGAAAAG-3′; IL-6 reverse, 5′-CTCTGAAGGACTCTGGCT-3′; IL-1β forward, 5′-CTTCAGGCAGGCAGTATC-3′; IL-1β reverse, 5′-GCTTTTTTGTTGTTCATCTC-3′; has-miR-93-5p, CAAAGUGCUGUUCGUGCAGGUAG; Universal primer, 5′-GCGAGCACAGAATTAATACGAC-3′. The PCR was set at the initial denaturation of 5 min at 95°C, following with 5 s at 95°C, 20 s at 72°C in a total of 40 cycles, and finally, 30 s at 95°C, 20 s at 58°C, and 30 s at 95°C.

### 2.4 Extraction and identification of ADSC-exos

Take the third-generation cells cultured in ADSCs and inoculate 5 × 10^6^ cells in each culture dish. After culturing overnight, discard the culture medium, wash twice with phosphate-buffered saline (PBS) (100–200 μL), culture with Exo-Clear cell Growth Medium (SBI, USA), and collect the cell supernatant for 24 h. A total of 4–5 supernatants in cell culture dishes were collected and centrifuged at 3,000 × *g* for 15 min, and cells/cell fragments were discarded. These supernatants were transferred to a new centrifuge tube, and exosome extraction reagent was added at a ratio of 4:1 supernatant to ExoQuick TC (SBI, USA) (volume ratio) and mixed and left overnight at 4°C. After centrifugation for 30 min at 1,500 × *g*, the supernatants were discarded, followed by centrifugation for 5 min at 1,500 × *g*. The supernatants were carefully absorbed and discarded, and the precipitations were exosomes. As previously reported [31], transmission electron microscope (TEM) could show purified-ADSC-exos double-layer capsule ultrastructure. In addition, biomarkers of exosome (including CD9, CD81, and TSG101) were detected by western blot assay.

### 2.5 Western blot assay

The protein expression of cells or tissues was detected by western blot assay as previously described [32]. In brief, cells and exosome samples were collected and homogenized using the RIPA buffer. Equal total proteins (30 µg/lane) were separated by sodium dodecyl sulfate–polyacrylamide gel electrophoresis (Bio-Rad, USA) and transferred onto the polyvinylidene fluoride membrane (Millipore, USA). Next, the membranes were blocked by 5% bovine serum albumin for 2 h at room temperature, followed by incubation with primary antibodies against at 4°C overnight. After washing with 1× PBS for three times, the membranes were incubated with HRP-conjugated secondary antibodies (1:5,000, ABclonal, China) for 2 h at room temperature. The bands were then developed using enhanced chemiluminescence chromogenic substrate (GE Healthcare, UK) and analyzed by the ImageJ software. The primary antibodies used were as follows: anti-CD9, CD81, and TSG101 antibody (1:1,000; ABclonal), anti-P-Akt473 antibody (1:1,000, 4,060; CST), anti-Akt antibody (1:1,000, 4,685; CST), anti-P-PIK3B antibody (1:1,000, AB182651; Abcam), anti-P-mTOR antibody (1:1,000, 5,536; CST), anti-mTOR antibody (1:1,000, 2,983; CST), anti-Bcl-2 antibody (1:1,000, a11025; ABclonal), anti-Bax antibody (1:1,000, A18642; ABclonal), anti-LC3-I/II antibody (1:1,000, A7198; ABclonal), and adopted GAPDH (1:1,000, AC002; ABclonal) as an internal control.

### 2.6 Dual luciferase reporter gene assay

Dual luciferase reporter gene assay corroborated the targeting relationships between ADAMTS9 and miR-93-5p. To generate psicheck2-ADAMTS9-WT and psicheck2-ADAMTS9-MUT vectors, the wild type (WT) containing the predicted target site and the mutant type (MUT) with the binding site deleted were amplified and cloned into the psicheck2 plasmid. Afterwards, the luciferase vectors were, respectively, transfected into HEK293T cells along with miR-93-5p mimic or mimic NC. After 24 h of transfection, following the manufacturer’s protocol, we measured the relative luciferase activity by normalizing the firefly luminescence to the Renilla luminescence using the Dual-Luciferase Reporter Assay System (Promega, Madison, WI, USA).

### 2.7 Detection of nitric oxide (NO) content

When the chondrocytes in arthritis of articular mouse models were successfully constructed, the cell supernatants were collected. The nitrite concentration was spectrophotometrically determined using Griess reagent (1% sulfanilamide and 0.1% naphthylethylenediamide in 5% phosphoric acid).

### 2.8 Monodansylcadaverine (MDC) staining

MDC staining was used as a tracer of autophagic vesicles for autophagy detection. Positive cells were colored in their perinuclear region, cellular autophagy was observed, and all acidic vacuoles were stained. Cell climbing sheets were prepared overnight for group treatments, and 0.05 mmol/L MDC (Shanghai, Huzheng, Industrial Co., Ltd.) was added to the water bath at 37°C for 15 min and washed three times with PBS, followed by immobilization with 4% paraformaldehyde for 15 min. Fluorescence microscope observation was then performed on an anti-fluorescence quenching slide to avoid light.

### 2.9 Flow cytometry

Rat articular chondrocytes with corresponding treatments of each group were stained with propidium iodide (PI, 50 mg/mL). After 24 h of treatment, the cells were digested with EDTA-free trypsin at 37°C for 5 min, and another addition of 1 mL culture medium with serum was added to terminate digestion. The cells were collected and centrifuged at 1,000 rpm for 10 min with the supernatant being discarded. PI (50 mg/mL) staining was conducted according to the provided kit (Invitrogen, USA) and cell cycle was detected by flow cytometer (BD, USA).

### 2.10 Statistical analyses

All experiments were repeated three times and the data were presented as the mean ± standard deviation using SPSS 18.0 (SPSS, inc.). One-way analysis of variance and *post hoc* Dunnett’s T3 test were performed to compare the differences among and between groups, respectively. *P* < 0.05 was considered to indicate a statistically significant result.

## 3 Results

### 3.1 Construction of a mouse chondrocyte model of arthritis by IL-1β

**Figure 1:**
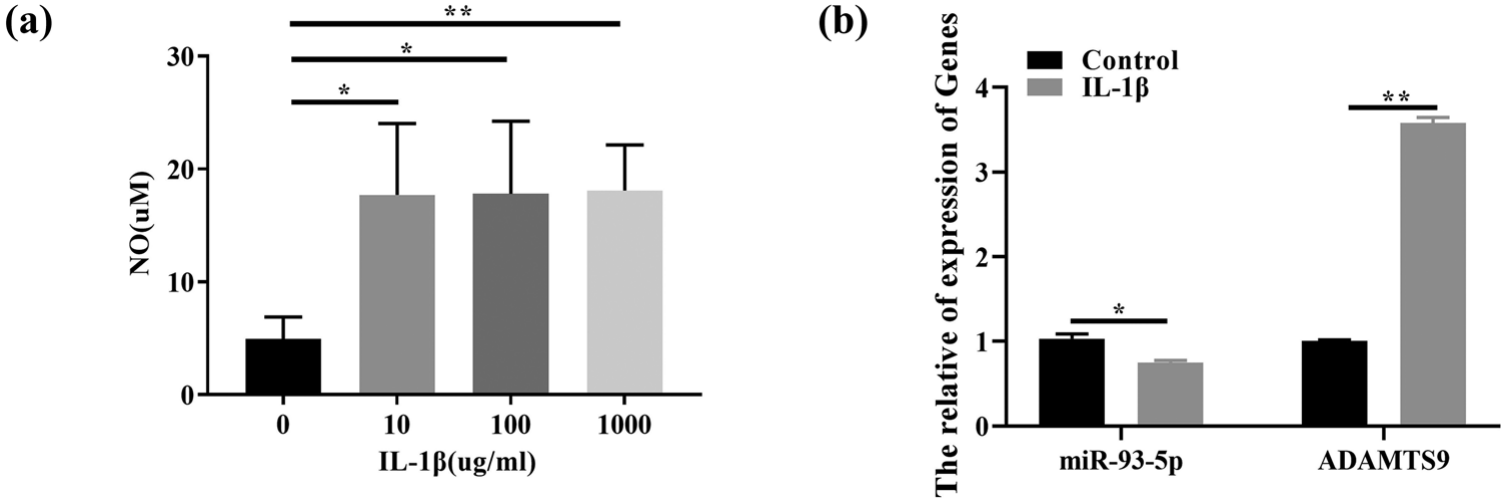
Construction of a mouse chondrocyte model of arthritis by IL-1β. Note: (a) when IL-1β concentration was 10 μg/mL, the articular chondrocyte model of arthritic mice was constructed. (b) RT-qPCR was performed to detect the expression of miR-93-5p and ADAMTS9 in IL-1β-induced chondrocytes. *P < 0.05; **P < 0.01.

When IL-1β concentration was 10 μg/mL (Figure 1a), the articular chondrocyte model of arthritic mice was constructed, and the expression of miR-93-5p in IL-1β induced chondrocytes was significantly decreased while ADAMTS9 was significantly increased (Figure 1b).

### 3.2 Validation of ADAMTS9 as a direct target gene of miR-93-5p

**Figure 2:**
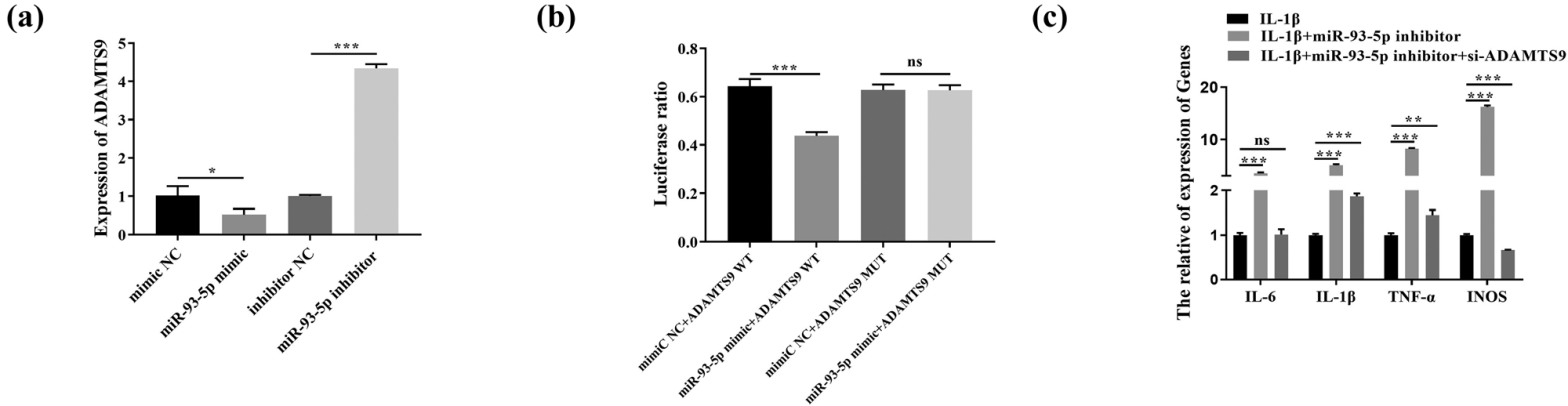
Validation of ADAMTS9 as a direct target gene of miR-93-5p. Note: (a) miR-93-5p mimic and miR-93-5p inhibitor were transfected into OA chondrocytes to overexpress and knockdown miR-93-5p, respectively. (b) The interaction between ADAMTS9 and miR-93-5p was detected with dual luciferase reporter gene assay. (c) RT-qPCR was performed to detect the expression of pro-inflammatory factors (IL-6, IL-1β, TNF-α) and iNOS. *P < 0.05; **P < 0.01; ***P < 0.001.

miR-93-5p mimic and miR-93-5p inhibitor were transfected into OA chondrocytes to overexpress and knock down miR-93-5p, respectively. As shown in Figure 2a, compared with the control group (mimic NC), the mRNA expression level of ADAMTS9 in the overexpression group (miR-93-5p mimic) was markedly downregulated. On the contrary, the expression level of ADAMTS9 in the knockdown group (miR-93-5p inhibitor) was dramatically upregulated compared with the control group (inhibitor NC). The interaction between ADAMTS9 and miR-93-5p was detected with dual luciferase reporter gene assay, which revealed targeted binding of miR-93-5p to ADAMTS9 and downregulation of ADAMTS9 expression (Figure 2b). Finally, the results of RT-qPCR are all demonstrated in Figure 2c, and silencing ADAMTS9 reversed the facilitation of miR-93-5p inhibitor on the expression of pro-inflammatory factors (IL-6, IL-1β, TNF-α) and iNOS.

### 3.3 miR-93-5p inhibited autophagy and apoptosis of IL-1β-treated chondrocytes by targeting ADAMTS9 to activate the PI3K/AKT/mTOR pathway

**Figure 3:**
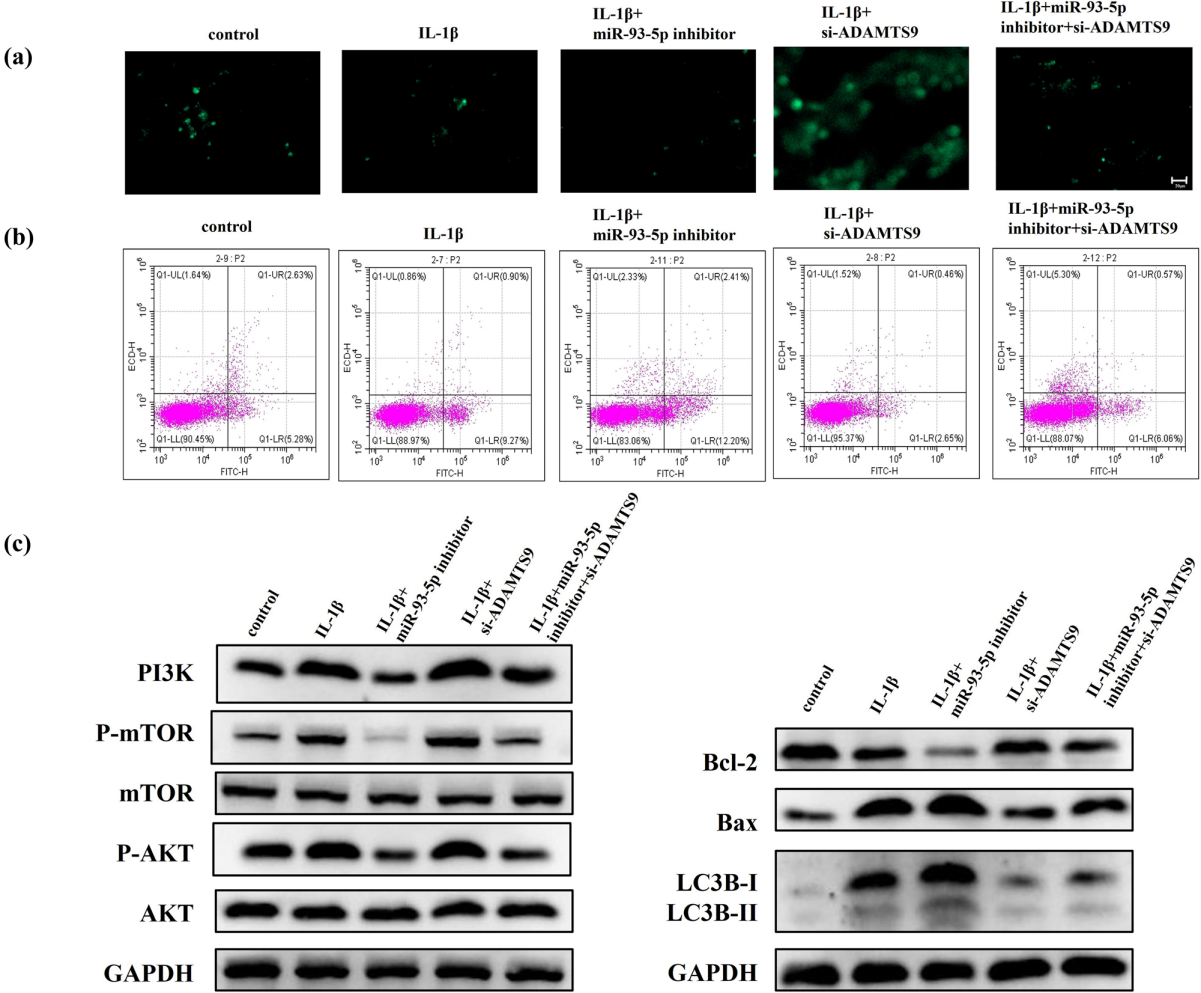
miR-93-5p inhibited autophagy and apoptosis of IL-1β-treated chondrocytes by targeting ADAMTS9 to activate the PI3K/AKT/mTOR pathway. Note: (a) MDC staining was used to detect the autophagy of control, IL-1β, IL-1β + miR-93-5p inhibitor, IL-1β + si-ADAMTS9, and IL-1β + miR-93-5p inhibitor + si-ADAMTS9 group in chondrocytes. (b) Flow cytometry was used to detect the apoptosis of control, IL-1β, IL-1β + miR-93-5p inhibitor, IL-1β + si-ADAMTS9, and IL-1β + miR-93-5p inhibitor + si-ADAMTS9 group in chondrocytes. (c) Western blot assay was used to detect the expression of PI3K, P-mTOR, P-AKT, Bcl-2/Bax, and LC3B-I/II expression of control, IL-1β, IL-1β + miR-93-5p inhibitor, IL-1β + si-ADAMTS9, and IL-1β + miR-93-5p inhibitor + si-ADAMTS9 group.

To analyze the contribution of miR-93-5p to the autophagy and apoptosis of IL-1β-treated chondrocytes, the cells were transfected with an miR-93-5p repressor and constructed a lentiviral vector silencing ADAMTS9. As shown in Figure 3a, autophagy chondrocytes were remarkably decreased after miR-93-5p inhibitor transfection in the condition of IL-1β treated, whereas silencing ADAMTS9 was the opposite. Finally, we conducted a functional rescue experiment. IL-1β-treated chondrocytes were simultaneously transfected with siRNA of ADAMTS9 (si-ADAMTS9) and miR-93-5p inhibitor to knock down both ADAMTS9 and miR-93-5p (si-ADAMTS9 + miR-93-5p inhibitor). The results showed that the inhibition of autophagy of miR-93-5p inhibitor could be partially reversed by si-ADAMTS9 in IL-1β-treated chondrocytes (Figure 3a). The results of flow cytometry are all demonstrated in Figure 3b; the inhibition effect of miR-93-5p inhibitor on apoptosis could be partially reversed by si-ADAMTS9 in IL-1β-treated chondrocytes. As determined by the western blot assay, the inhibition of miR-93-5p inhibitor on PI3K, P-mTOR, P-AKT, Bcl-2/Bax and LC3B-I/II expressions could be partially reversed by si-ADAMTS9 in IL-1β-treated chondrocytes (Figure 3c).

### 3.4 miR-93-5p derived from ADSC-exos inhibited autophagy and apoptosis of IL-1β-treated chondrocytes

**Figure 4:**
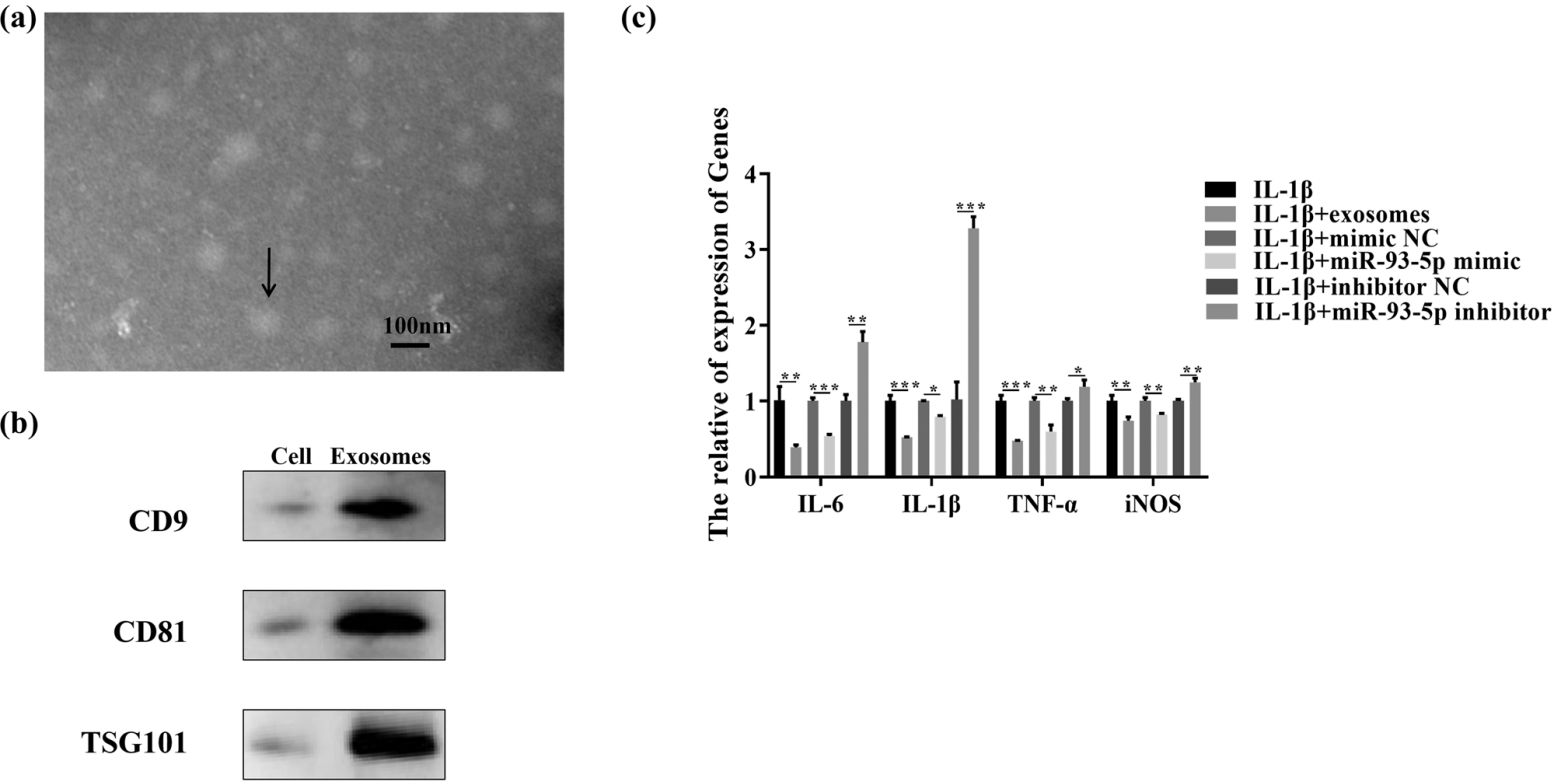
miR-93-5p derived from ADSC-exos inhibited autophagy and apoptosis of IL-1β-treated chondrocytes. Note: (a) the structure of isolated exosomes was analyzed by TEM. (b) Exosome markers CD9, CD81, and TSG101 in ADSC-exos were characterized by the western blot assay. *P < 0.05; **P < 0.01; ***P < 0.001. (c) RT-qPCR was performed to detect the expression of pro-inflammatory factors (IL-6, IL-1β, TNF-α) and iNOS in IL-1β, IL-1β + exo, IL-1β + exo + inhibitor/mimic NC, and IL-1β + exo + miR-93-5p inhibitor/mimic group. *P < 0.05; **P < 0.01; ***P < 0.001.

Recent studies have shown that ADSC-exos play an important role in OA [28,29]. To further clarify the protective effect of ADSC-exos after OA, we isolated and identified exosomes from ADSCs. TEM analysis of isolated exosomes showed round structures with diameters between 30 and 150 nm (Figure 4a). As shown in Figure 4b, exosome markers CD9, CD81, and TSG101 in ADSC-exos were characterized by the western blot assay. Previous study confirmed that miR-93-5p-enhanced ADSC-derived exosomes can prevent cardiac injury by targeting Atg7 and Toll-like receptor 4 (TLR4) and inhibiting hypoxia-induced autophagy and inflammatory cytokine expression [26]. To further clarify the protective effect of ADSC-exos after OA, we co-incubated ADSC-exos with the IL-1β-treated chondrocytes. As shown in Figure 4c, compared with IL-1β-treated chondrocytes, the expression of pro-inflammatory factors (IL-6, IL-1β, TNF-α) and iNOS in the experimental group (IL-1β + exosomes and IL-1β + miR-93-5p mimic) was significantly downregulated, while significantly upregulated in the IL-1β + miR-93-5p inhibitor group.

## 4 Discussion

In this study, miR-93-5p inhibited the autophagy and apoptosis of IL-1β-treated chondrocytes by targeting ADAMTS9 to activate the PI3K/AKT/mTOR pathway. Furthermore, ADSC-derived exosomal miR-93-5p inhibited autophagy and apoptosis of IL-1β-treated chondrocytes. Therefore, this study first suggested that the ADSC-exos/miR-93-5p/ADAMTS9 axis represents a promising therapeutic strategy for OA.

OA is known as a progressive degeneration of articular cartilage, resulting in the abnormal metabolism of subchondral bone osteoblast [33]. Although both pharmacologic therapies administrated intra-articular, orally and topically and non-pharmacologic treatments including exercise have been applied in OA therapy, the efficacy has been short-lived or not immediately [34]. At present, OA is mainly diagnosed by radiography, computed tomography, ultrasound, and magnetic resonance imaging. Using current methods, OA is usually diagnosed at a late stage of disease development, meaning that the lesions in the joint tissues have progressed to irreversible damage, missing the best time for intervention [35]. The development of new biomarkers may facilitate early detection of the disease and subsequent more effective treatment [36]. Abnormal regulation of miRNA levels is closely related to many diseases including OA [37,38]. Consequently, it is crucial to reveal genetic networks modulated via these miRNAs in OA and develop a new treatment method to suppress inflammation after OA.

miR-93, a member of the miR-106b-25 family, located within an intron of MCM7 gene, is highly expressed in a variety of cancers and acts as an oncogene [39]. Previous studies found that BMSC-exos exert a protective role in wound healing by upregulating miR-93-3p [40]. Moreover, miR-93-5p is involved in the inflammatory cytokine expression and autophagy [25,26]. miR-93-5p has been revealed to regulate IL-1β-induced cartilage degradation and chondrocyte apoptosis in OA by targeting TCF4 [27]. The previous investigation has found that IL-1β plays a critical role in cartilage degeneration, which is associated with the etiology of OA [41]. According to available studies, IL-1β is increased in osteoarthritic joint tissues and contributes to the development of OA. And it was able to induce the decrease of the generation of collagen type II and aggrecan and increased the production of MMPs [42]. Precious studies identified two aggrecanase proteins, ADAMTS-4 and ADAMTS-5 [43,44]. They are both the members of the ADAMTS family of zinc metalloproteinases. The analysis of gene expression and methylation datasets identified ADAMTS9 as biomarkers for OA [12,45]. In this study, we found that the expression of miR-93-5p in IL-1β induced chondrocytes was significantly decreased, while ADAMTS9 was significantly increased. The dual luciferase reporter gene assay showed that ADAMTS9 was the target gene of miR-93-5p. And then, we testified that the inhibition of autophagy and apoptosis of miR-93-5p inhibitor could be partially reversed by si-ADAMTS9 in IL-1β-treated chondrocytes. According to available evidence, the PI3K/AKT/mTOR signaling pathway plays a key role in cartilage degradation, subchondral bone dysfunction, and synovial inflammation and participates in the development of OA [46]. Previous research has also indicated that the PI3K/AKT/mTOR pathway may be associated with various aspects of autophagy [47,48]. And several studies have demonstrated that the PI3K/Akt/mTOR signaling pathway is closely related to the autophagy and apoptosis of IL-1β-treated articular chondrocytes [49,50,51]. In this study, PI3K expression was found to be decreased following the inhibition of miR-93-5p in IL-1β-treated chondrocytes, along with the increase of apoptosis and autophagy. In addition, the inhibition of miR-93-5p inhibitor on PI3K, P-mTOR, and P-AKT expressions could be partially reversed by si-ADAMTS9. Taken together, the results suggested that miR-93-5p may considerably inhibit the development of OA by modulating the PI3K/AKT/mTOR pathway.

Recent studies have shown that ADSC-exos play an important role in OA [28,29]. For example, ADSC-exos increased Prdx6 expression in OA chondrocytes stimulated with IL-1β- and Prdx6-protected OA chondrocytes against the consequences of IL-1β stimulation [52]. ADSC-exos inhibited the inflammation and promoted chondrogenesis by upregulating miR-145/miR-221 [53]. In our study, the expression of pro-inflammatory factors (IL-6, IL-1β, TNF-α) and iNOS in the experimental group (IL-1β + exosomes and IL-1β + miR-93-5p mimic) was significantly downregulated, while significantly upregulated in the IL-1β + miR-93-5p inhibitor group.

## 5 Conclusion

In summary, within the scope of our understanding, this study first demonstrated that ADSC-derived exosomal miR-93-5p inhibited the autophagy and apoptosis of IL-1β-treated chondrocytes through PI3K/AKT/mTOR signaling pathway, thereby inhibiting inflammation and alleviating OA. This provides a new method for the treatment of OA.

## Abbreviations

OA: osteoarthritis
MDC: monodansylcadaverine
TEM: transmission electron microscopy
ADAMTS9: ADAM metallopeptidase with thrombospondin type 1 motif, 9
siRNA: small-interfering RNA
RT-qPCR: reverse transcription quantitative polymerase chain reaction
GAPDH: glyceraldehyde 3-phosphate dehydrogenase
NC: negative control
WT: wild type
MUT: mutant type
DMEM: Dulbecco’s modified eagle medium
SDS-PAGE: Sodium dodecyl sulfate–polyacrylamide gel electrophoresis
PVDF: Polyvinylidene fluoride
BSA: Bovine serum albumin
